# Driving the conversion of phytosterol to 9α-hydroxy-4-androstene-3,17-dione in *Mycolicibacterium neoaurum* by engineering the supply and regeneration of flavin adenine dinucleotide

**DOI:** 10.1186/s13068-023-02331-1

**Published:** 2023-06-08

**Authors:** Lu Song, Jie Ke, Zhi-Kun Luo, Liang-Bin Xiong, Yu-Guo Dong, Dong-Zhi Wei, Feng-Qing Wang

**Affiliations:** 1 State Key Laboratory of Bioreactor Engineering, Newworld Institute of Biotechnology, East China University of Science and Technology, Shanghai, 200237 China; 2 Jiading District Central Hospital Affiliated Shanghai University of Medicine and Health Sciences, Shanghai, 201800 China; 3 Key Laboratory of Biocatalysis and Intelligent Manufacturing (ECUST), China National Light Industry, Shanghai, 200237 China

**Keywords:** Cofactor regeneration, Flavin adenine dinucleotide, *Mycolicibacterium*, Steroid synthons, Sterols

## Abstract

**Background:**

The conversion of phytosterols to steroid synthons by engineered *Mycolicibacteria* comprises one of the core steps in the commercial production of steroid hormones. This is a complex oxidative catabolic process, and taking the production of androstenones as example, it requires about 10 equivalent flavin adenine dinucleotide (FAD). As the high demand for FAD, the insufficient supply of FAD may be a common issue limiting the conversion process.

**Results:**

We substantiated, using the production of 9α-hydroxy-4-androstene-3,17-dione (9-OHAD) as a model, that increasing intracellular FAD supply could effectively increase the conversion of phytosterols into 9-OHAD. Overexpressing *ribB* and *ribC*, two key genes involving in FAD synthesis, could significantly enhance the amount of intracellular FAD by 167.4% and the production of 9-OHAD by 25.6%. Subsequently, styrene monooxygenase *NfStyA2B* from *Nocardia farcinica* was employed to promote the cyclic regeneration of FAD by coupling the oxidation of nicotinamide adenine dinucleotide (NADH) to NAD^+^, and the production of 9-OHAD was further enhanced by 9.4%. However, the viable cell numbers decreased by 20.1%, which was attributed to sharply increased levels of H_2_O_2_ because of the regeneration of FAD from FADH_2_. Thus, we tried to resolve the conflict between FAD regeneration and cell growth by the overexpression of catalase and promotor replacement. Finally, a robust strain NF-P2 was obtained, which could produce 9.02 g/L 9-OHAD after adding 15 g/L phytosterols with productivity of 0.075 g/(L h), which was 66.7% higher than that produced by the original strain.

**Conclusions:**

This study highlighted that the cofactor engineering, including the supply and recycling of FAD and NAD^+^ in *Mycolicibacterium*, should be adopted as a parallel strategy with pathway engineering to improve the productivity of the industrial strains in the conversion of phytosterols into steroid synthons.

**Supplementary Information:**

The online version contains supplementary material available at 10.1186/s13068-023-02331-1.

## Background

**Fig. 1:**
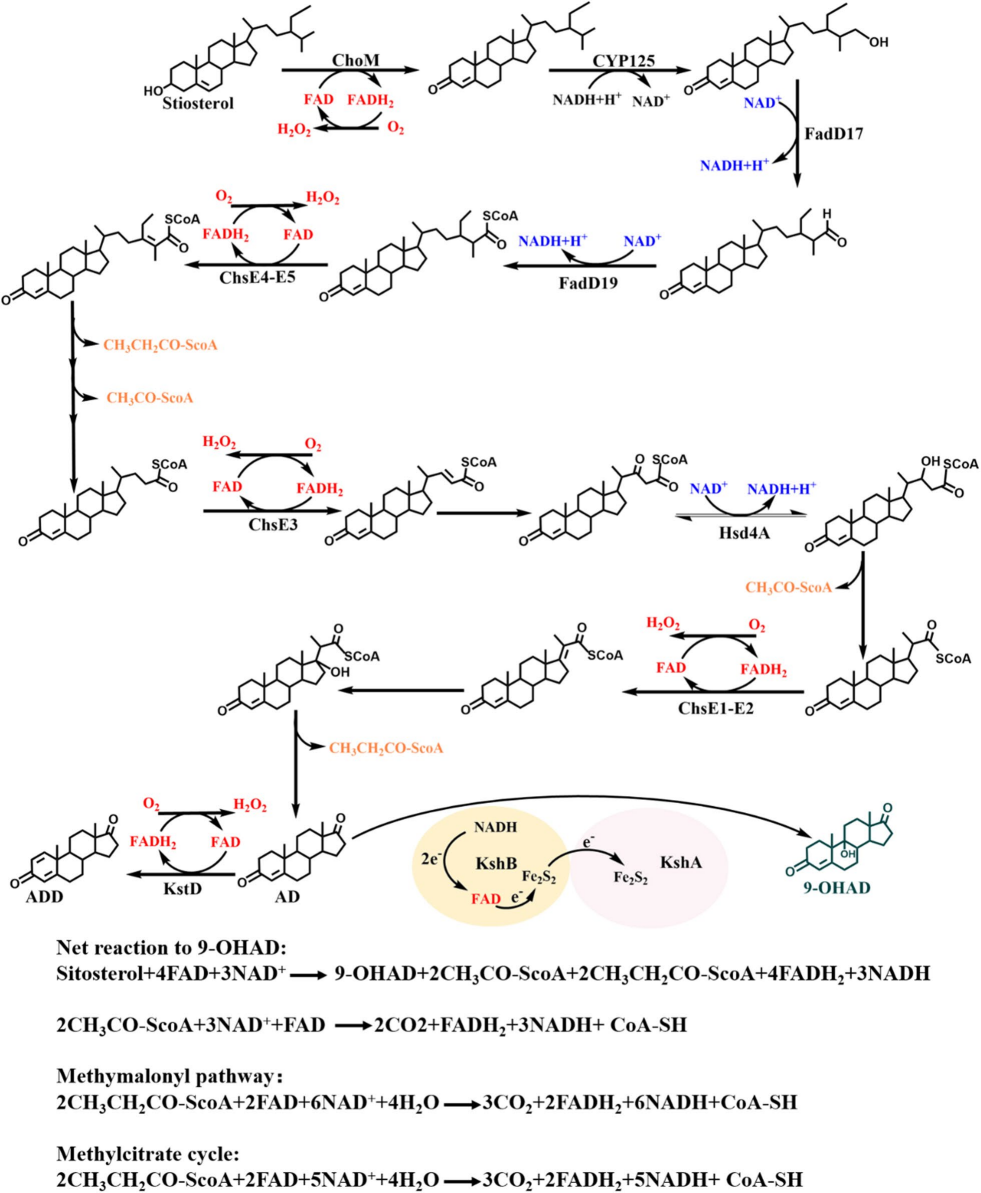
Schematic profiles of the catabolism of sterol side-chain. Sterols share a conserved degradation pathway. Thus, β-sitosterol was used as a model substrate to depict the route of sterols to target product 9-OHAD (in green color). This process involved multi-step NAD^+^-dependent (in blue color) and FAD-dependent enzymatic reactions (in red color). The re-oxidation of FAD depended on oxygen. Oxygen acted as an electron acceptor and was reduced to H_2_O_2_ during the reaction. Further, the metabolism of acetyl-CoA and propionyl-CoA (in orange color) also involved the utilization of FAD. Calculations of the NAD^+^ and FAD requirement and the generation of NADH and FADH_2_ during the conversion of sterols into 9-OHAD are listed at the bottom. 9-OHAD, 9a-hydroxy-androst-4-ene-3,17-dione; AD, androst-4-ene-3, 17-dione; ADD, androst-1,4-diene-3,17-dione

Steroids belong to the second largest category drug and have a wide range of clinical therapeutic uses [1]. Microbial conversions are playing irreplaceable roles in the synthesis of such drugs, among which converting cheap phytosterols by *Mycolicibacterium* species to obtain C19 and C22 steroidal metabolites is one of the most important steps, as they have been widely used as the value-added synthons of diverse steroid hormones in the industry [2]. The conversion of phytosterols into these synthons has been well documented [3–11]. Based on this, the steroids can be generated by modifying the complex catabolism pathway of sterols in *Mycolicibacterium* species (Fig. 1). To date, some consensus engineering strategies have been proposed to develop different synthon-producing strains and to improve their productivities. A classic strategy is to block the competition and degradation routes by gene inactivation and augment the rate-limiting steps by gene overexpression, and the other strategy is to enhance the metabolic flow of sterols by manipulating regulatory factors [5, 6] and increasing the transport efficiency of sterols from outside to inside the cell [10, 11]. Further, engineering the supply and regenerating redox cofactors involved in the catabolic process of sterols were essential to drive the conversion of phytosterols into steroid synthons [12–15].

The catabolism of sterols is a process of oxidative degradation with generation of multiple reducing equivalent. For instance, approximately 17 equivalents nicotinamide adenine dinucleotide (NADH) and 9 equivalents flavin adenine dinucleotide (FADH_2_) can be generated, in theory, during the conversion process from sitosterol to 9-OHAD [9, 12, 16], if the formed metabolites acetyl-coenzyme A (CoA) and propionyl-CoA can be completely degraded to CO_2_ and H_2_O [13, 17] as well as the key 9α-hydroxylase is functionalized by FAD transporting electrons from ferredoxin reductase KshB to terminal oxygenase KshA in the 9α-hydroxylation of steroids [18, 19] (Fig. 1). Although the NADH-reducing equivalent is not directly produced during the conversion of sitosterol into 9-OHAD, regulating the intracellular ratio of NAD^+^/NADH has been demonstrated as effective in increasing the productivity of C19 synthons, such as androst-4-ene-3,17-dione (AD) [12, 20], androst-1,4-diene-3,17-dione (ADD) [14], 9,21-dihydroxy-20-methyl-pregna-4-en-3-one (9-OH-HP) [15]. In fact, besides the generation of NADH, the mass formation of FADH_2_ is an important feature of the catabolism of sterols in *Mycobacteria*. This is because FAD is the crucial cofactor of multiple key enzymes involved in the catabolism of phytosterols, such as cholesterol oxidase ChoM, acyl-CoA dehydrogenase ChsE4-ChsE5, ChE3, ChsE1-ChsE2, and 3-ketosteroid-Δ^1^-dehydrogenase KstD, and is directly produced in the catabolic process (Fig. 1) [21]. Taking the conversion of sitosterol to 9a-hydroxy-androst-4-ene-3,17-dione (9-OHAD) as an example, four equivalents of FADH_2_ are directly generated in the process and five equivalents of FADH_2_ are indirectly produced when the intermediates acetyl-CoA and propionyl-CoA. The further complete catabolism of acetyl CoA (acetate) and propionyl CoA (propionate) are digested completely through different pathways in *Mycolicibacterium* [22] (Fig. 1). The biased use of FAD in the catabolism of phytosterols confers some significant effects on the physiological metabolism of mycobacterial cells, especially when the metabolic flux is boosted. A typical manifestation is that serious growth inhibition of mycobacterial cells occurs along with the conversion of phytosterols, which is partly attributed to the excess generation of hydrogen peroxide (H_2_O_2_) in the cells as a byproduct of FAD regeneration from FADH_2_ (Fig. 1). The excess accumulation of H_2_O_2_ inevitably shows the significant toxic effect on the growth and metabolism of mycobacterial cells, consequently attenuating the conversion of sterols into target synthons. Thus, neutralizing the excessive H_2_O_2_ in mycobacterial cells by overexpressing catalase (CAT), as well as augmenting the synthesis of antioxidants MSH and EGT have been affirmed to be an effective strategy to promote the conversion of sterols into steroid synthons [9]. Nevertheless, the specific effects of the supply and regeneration of FAD on the biotransformation of phytosterols into steroid synthons have not been investigated earlier.

Since FAD is an essential cofactor involved in the catabolism of phytosterols (Fig. 1), the supply and cyclic regeneration of FAD might be a key factor influencing the bioconversion of phytosterols into target products. In prokaryotes, FAD is mainly synthesized from two major precursors guanosine-5′-triphosphate (GTP) and ribulose-5-phosphate (R5P) with the concerted catalysis of six enzymatic reactions encoded by *ribA*, *ribB*, *ribD*, *ribH*, *ribC* and *ribF* [23–26]. The overexpression of *FMN1* gene encoding riboflavin kinase RibF in *Candida famata* could lead to a 400-fold increase in the production of flavin mononucleotide (FMN) [23], and replacing the promoter of *FMN1* with a stronger constitutively promoter could also achieve 14-fold improvement in FAD production [26]. Hence, augmenting the FAD synthetic pathway may help increase the intracellular supply of FAD in mycobacterial cells, thereby driving the conversion of phytosterols into steroid synthons as a chain effect of the increased FAD levels. However, he excessive consumption of FAD can accelerate the re-oxidation of FADH_2_, leading to the over-generation of H_2_O_2_ as well as other reactive oxygen species (ROS), such as peroxide anion (O_2_^−^), which seriously impacts a wide range of cellular physiological and biochemical processes [9]. Hence, the intracellular FAD level, as well as the ROS level, should be comprehensively regulated in the engineered phytosterol-transforming *Mycobacteria* to boost the conversion of phytosterols into target synthons.

In this study, the enhanced FAD synthesis and cyclic regeneration were investigated in a model of the conversion of phytosterols into 9-OHAD by *Mycolicibacterium neoaurum* to evaluate the specific effects of FAD supply on the conversion of phytosterols into steroid synthons [3]*.* As FMN is the direct precursor for FAD synthesis, it was first added extracellularly to assess its effect on the transformation of phytosterols into 9-OHAD. The results confirmed that productivity could be significantly improved by adding FMN in an appropriate amount. Thus, the intracellular FAD level was enhanced by engineering the biosynthetic pathway of FAD to evaluate the effects on 9-OHAD production. As the productivity of 9-OHAD was significantly enhanced along with the increase in intracellular FAD level, a strategy to promote the cyclic regeneration of FAD from FADH_2_ was then tested by coupling the oxidation of NADH to NAD^+^ with a styrene monooxygenase from *N. farcinica*. Simultaneously, the *CAT* gene encoding catalase was overexpressed to neutralize the excessive H_2_O to eliminate the toxic effect of H_2_O_2_ generated from the FAD regeneration. The promoter replacement was tried to harmonize FAD regeneration and cell growth. Finally, a robust strain NF-P2 was developed, the 9-OHAD productivity of which was enhanced by 66.7% compared with that of the original strain, clearly demonstrating that engineering the supply and regeneration of FAD was an effective and practical way to drive the conversion of phytosterols into steroid synthons by engineered *Mycolicibacterium.*

## Results

### Evaluating the effects of intracellular FAD levels on the conversion of phytosterols

**Fig. 2:**
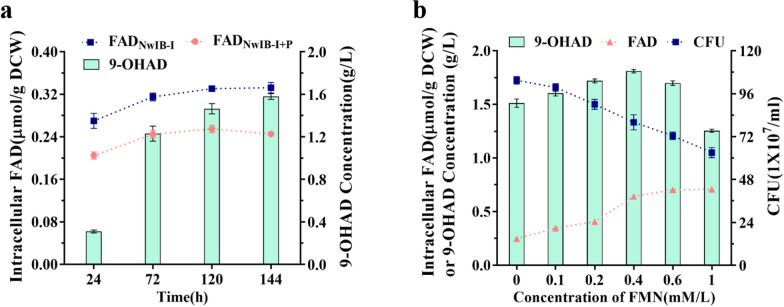
Analysis of FAD levels in the conversion of phytosterols into 9-OHAD. **a** Time profile of the intracellular FAD concentration in strain NwIB-I with or without adding phytosterols. **b** Effects of adding different concentrations of FMN on intracellular FAD level, cell growth, and 9-OHAD production in strain NwIB-I. Mycolicibacterial strains were cultivated in fermentation medium containing 4 g/L phytosterols at 30 °C for 144 h. The data represent mean ± standard deviation of three measurements

Although FAD has long been characterized as an essential coenzyme directly involved in the catabolism of sterols in *Mycobacterium* species (Fig. 1), no study has been conducted to evaluate its effects on the conversion of phytosterols into steroid synthons for biotechnological applications. In this study, the dynamic change in FAD level along with the transformation of phytosterols into 9-OHAD was first profiled in a typical 9-OHAD-producing strain *M. neoaurum* NwIB-I [3] to investigate its effect on the conversion process. The results showed that, along with the conversion of phytosterols, the intracellular FAD concentration decreased rapidly, and was much lower than the control without adding phytosterol (Fig. 2a). This indicated that a large amount of FAD was consumed in the conversion process of phytosterols to 9-OHAD, and the regeneration of FAD was not effective enough to maintain its physiological level during the conversion process. Obviously, the reduced FAD level was not conducive to promote the conversion from phytosterols to 9-OHAD as well as the other target synthons.

As FMN is the direct precursor of FAD biosynthesis catalyzed by the FAD synthase [27], FMN supplement may be beneficial to increase the intracellular FAD levels during the conversion process of phytosterols [12]. Exogenous FMN was added to the culture of NwIB-I with phytosterols to further evaluate the effect of increased FAD level on the conversion of phytosterols. The results indicated that the intracellular FAD level was significantly enhanced by 2.4-fold from 0.26 μmol/g DCW to 0.64 μmol/g DCW with the increase in FMN addition to 0.4 mmol/L, and the production of 9-OHAD was significantly enhanced by about 20% (Fig. 2b). However, when more than 0.4 mM/L FMN was added, FAD level no longer increased accordingly, and the production of 9-OHAD was greatly reduced. This might be partly attributed to the toxicity of FMN addition on cell survival, as along with the number of living cells decreased linearly after adding FMN. For example, the number of living cells dropped by 22.3% and 38.8%, respectively, when 0.4 mmol/L and 1.0 mmol/L FMN was added. Hence, the enhanced FAD level by moderate FMN addition (< 0.4 mmol/L) was beneficial to the conversion of phytosterols. The addition of FMN showed high toxicity to the cells, thus compromising the conversion capacity of phytosterols to 9-OHAD at higher concentrations. Therefore, it was necessary to take appropriate measures to regulate the synthesis of FAD and reduce metabolic toxicity to promote the conversion of phytosterols into 9-OHAD as well as other steroids.

### Enhancing intracellular biosynthesis of FAD to enhance the conversion of phytosterols

**Fig. 3:**
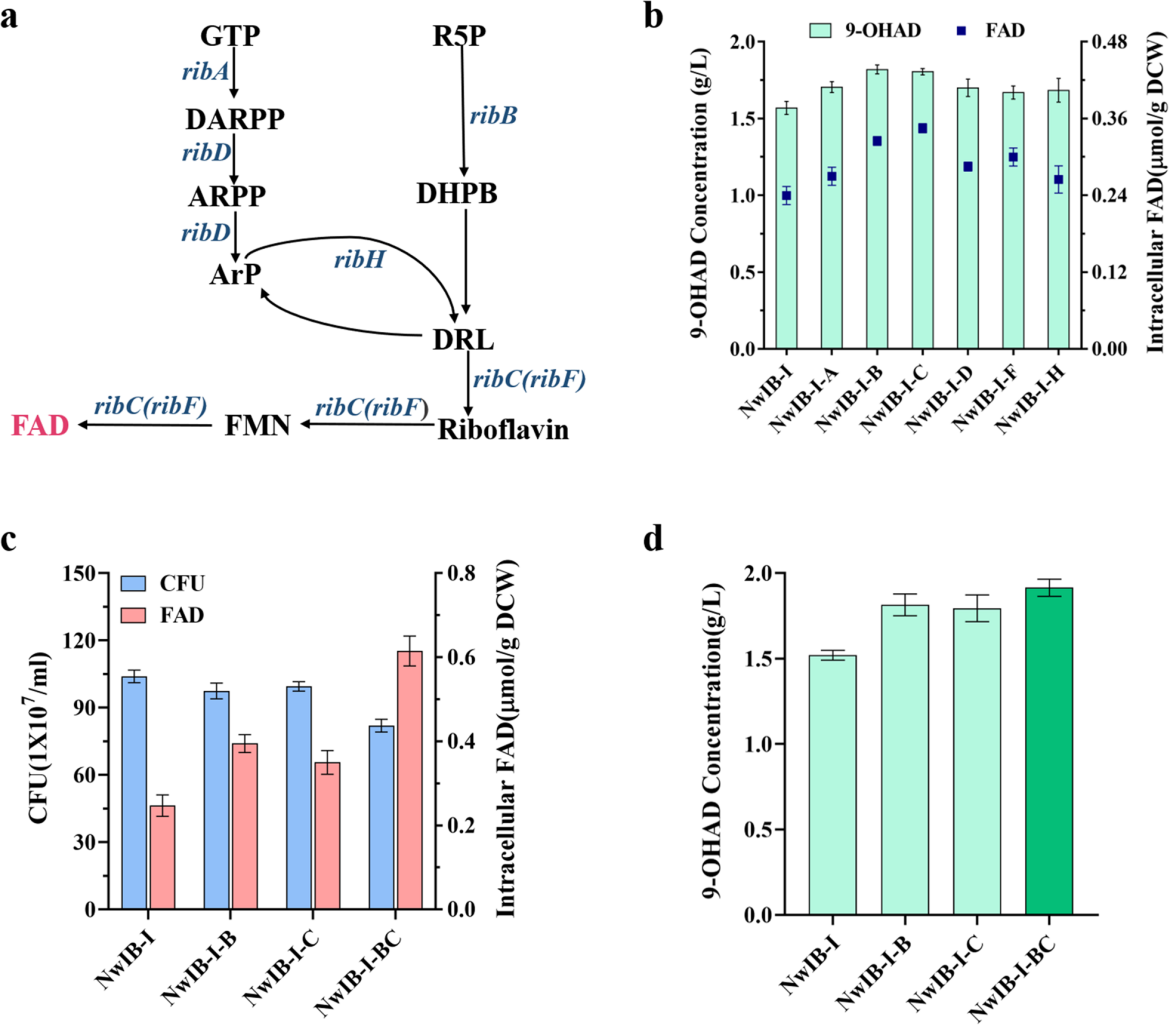
FAD supply in engineered 9-OHAD-producing strains. **a** Schematic illustration of the biosynthesis pathway of FAD production in prokaryotes. Enzymes encoded by the genes were as follows: *ribA*, GTP cyclohydrolase II; *ribB*, 3,4-dihydroxy-2-butanone 4-phosphate synthase; *ribD*, fused pyrimidine deaminase/uracil reductase; *ribH,* lumazine synthase; *ribF*, riboflavin synthase; *ribC*, riboflavin kinase/FMN adenylyltransferase. The metabolic intermediates were as follows: R5P, ribulose-5-phosphate; DHBP, ribulose-5-phosphate; ARPP, 5-amino-6-(5-phospho-D-ribitylamino)-uracil; Arp, 5-amino-6-(D-ribitylamino) uracil; DARPP, 2,5-diamino-6-ribosylamino-4(3H)-pyrimidinone 5’-phosphate; GTP, guanosine-triphosphate; DRL, 6,7-dimethyl-8-(1-D-ribityl) lumazine. **b** Intracellular FAD concentration and 9-OHAD production in engineered strains in which *ribA*, *ribD*, *ribB, ribH*, *ribC*, and *ribF* was overexpressed individually. **c** Intracellular FAD concentration and cell growth and **d** 9-OHAD production in engineered strains with combined overexpression of *ribB* and *ribC*. All engineered strains were cultivated in fermentation medium containing 4 g/L phytosterols at 30 °C for 144 h. The data represent the mean ± standard deviation of three measurements

The biosynthesis flux of FAD was improved by metabolic engineering to directly determine the effect of the enhanced intracellular FAD level on the conversion of phytosterols. Among microorganisms, the biosynthesis of FAD was carried out by two major precursors: GTP and R5P via a pathway involving six enzymatic reactions encoded by six *rib* genes (Fig. 3a) [25]. Each of the key genes involved in FAD synthesis, including *ribA*, *ribB*, *ribC*, *ribD*, *ribF*, and *ribH*, was augmented in strain NwIB-I to enhance the biosynthesis of FAD, obtaining strains NwIB-I-A, NwIB-I-B, NwIB-I-C, NwIB-I-D, NwIB-I-F, and NwIB-I-H, respectively. The results indicated that all of the overexpressed gene could significantly enhance the intracellular FAD levels and the production of 9-OHAD (Fig. 3b). Among these genes, *ribB* and *ribC* were most important and were combinatorially overexpressed to further promote the synthesis of FAD and the conversion of phytosterols into 9-OHAD. As expected, the resulted strain NwIB-BC displayed further enhancement in intracellular FAD level and the production of 9-OHAD compared to the strain NwIB-I, NwIB-I-B and NwIB-I-C (Fig. 3c, d). Taking the results after 144 h as examples, the intracellular FAD level in strain NwIB-BC has been improved by 158.3% from 0.24 μmol/g DCW to 0.62 μmol/g DCW and the production titer of 9-OHAD was improved by 25.6% from 1.52 to 1.91 g/L. These results showed that intracellular FAD level or FAD supply does play a crucial role in the conversion of phytosterols to steroid synthons, and also displayed a significant toxic effect on cell survival, as the number of living cells dropped by 21.1% comparison with strain NwIB-I at 144 h (Fig. 3c). Subsequently, the strain NwIB-BC was used as an FAD-enhanced strain for further investigation.

### Promoting the combined cyclic regeneration of FAD and NAD^+^

During the catabolism of phytosterols, dozens of reduced cofactors NADH and FADH_2_ were generated (Fig. 1). If these cofactors cannot be recycled in time, the catabolism of phytosterols was attenuated, which was not conducive to the conversion of phytosterols to steroid synthons. Previous studies suggested that promoting the regeneration from NADH to NAD^+^ can effectively boost the productivity of some steroidal synthons in *Mycolicibacterium*, such as AD and ADD [12, 14, 20]. In fact, most of NADH is generated though respiratory chain via an oxidative process from FAD to FADH_2_ under normal physiological conditions. However, no attempts were made to enhance the conversion of phytosterols into valuable products by promoting the cyclic regeneration of FAD from FADH_2_. Therefore, an auxiliary system for the simultaneous regeneration of FAD and NAD^+^ was developed in strain NwIB-BC by introducing an NADH-FAD(H_2_) dependent styrene monooxygenase.

**Fig. 4:**
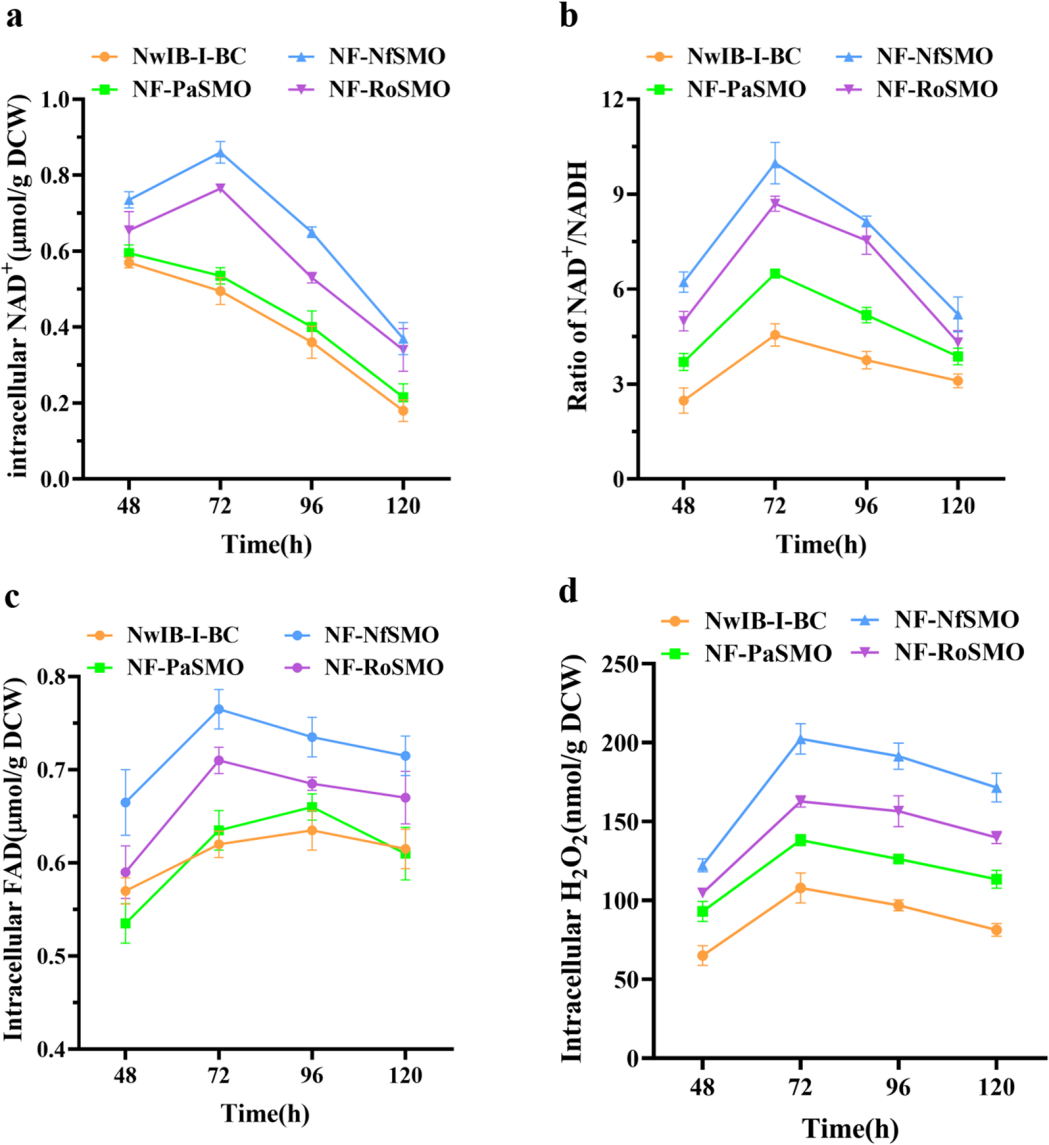
Effects of overexpressing the flavin-containing monooxygenase on **a** intracellular FAD level, **b** NAD^+^ level, **c** NADH/NAD^+^ ratio, and **d** H_2_O_2_ level. All engineered strains were cultivated in fermentation medium containing 4 g/L phytosterols at 30 °C for 144 h and the fermentation culture was collected regularly at 48–120 h to measure intracellular FAD, NAD^+^, NADH and H_2_O_2_. The data represent the mean ± standard deviation of three measurements

**Fig. 5:**
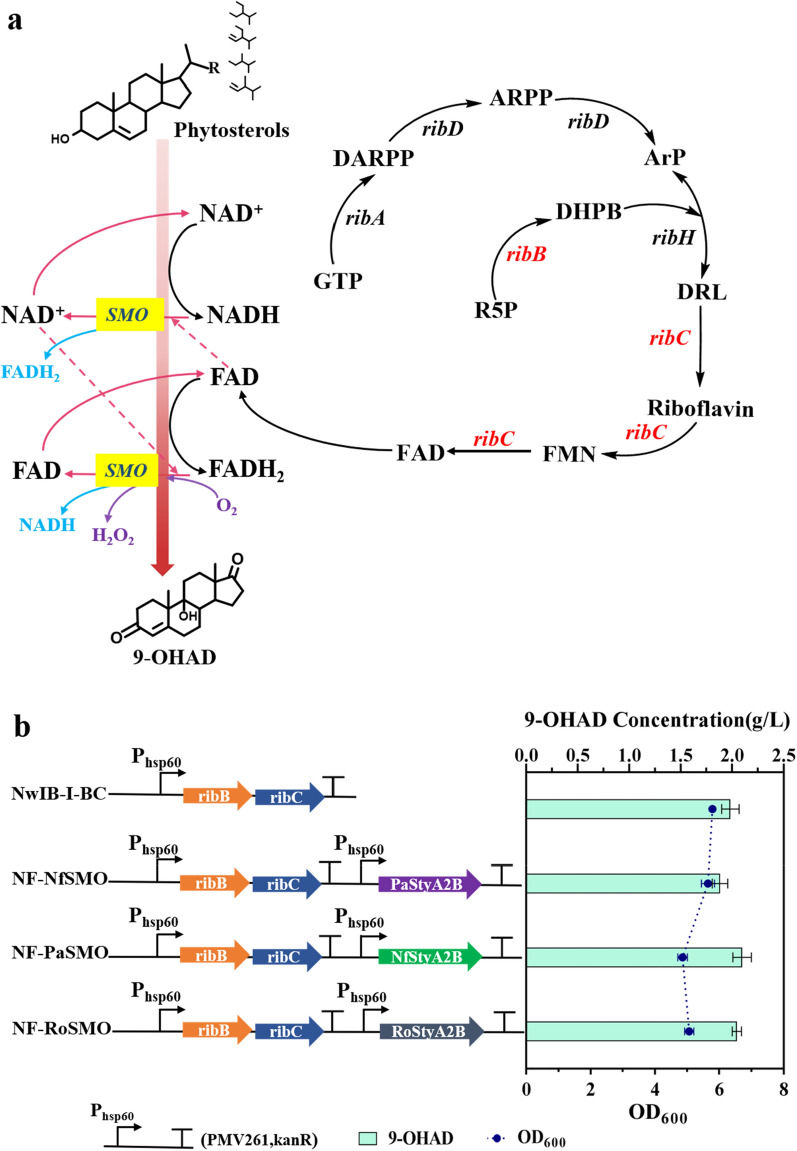
Cyclic regeneration of FAD and NAD^+^. (**a**) Schematic illustration of the cyclic regeneration of FAD and NAD^+^. The conversion of phytosterols into 9-OHAD was accompanied by the transformation of NAD^+^ into NADH and FAD into FADH_2_ in *Mycolicibacterium*. The combined expression of key genes *ribB* and *ribC* in FAD biosynthesis pathway resulted in the supply of intracellular FAD. The NADH-FAD(H2)-dependent styrene monooxygenase SMO (in yellow color) was expressed to promote the cyclic regeneration of FAD and NAD^+^ including the catalytic mechanism of the self-sufficient SMO. FAD regeneration was performed by the NAD^+^ (in red dashed) and oxygen (in solid purple line) consumption and production of NADH (in blue) and H_2_O_2_ (in purple), respectively. NAD^+^ regeneration was performed by the FAD consumption (in red dashed) and FADH_2_ production (in solid blue line). (**b**) Effect of overexpressing the flavincontaining monooxygenase genes *RoStyA2B* (GenBank: ANS29975.1), *PaStyA2B* (GenBank: ABM10099.1), and *NfStyA2B* (GenBank: BAD56094.1) on 9-OHAD production and cell growth; All engineered strains were cultivated in fermentation medium containing 4 g/L phytosterols at 30℃ for 144 h. The data represent the mean ± standard deviation of three measurements. The data represent the mean ± standard deviation of three measurements.

In this study, three genes, including *RoStyA2B* from *R. opacus* 1CP [28], *NfStyA2B* from *N. farcinica*, and *PaStyA2B* from *Paenarthrobacter aurescens* were tested, generating strains NF-NfSMO, NF-RoSMO, and NF-PaSMO, respectively. All of the strains showed significant changes in the contents of FAD and NAD^+^, demonstrating that all of these styrene monooxygenases had an oxidative effect on NADH and FADH_2_ as expected. The strain NF-NfSMO performed best in the regeneration of FAD and NAD^+^, resulting in an 89.3% increase in the content of NAD^+^ (Fig. 4a), 23.3% increase in the content of FAD (Fig. 4c), and 1.22-fold increase in the ratio of NAD^+^/NADH (Fig. 4b) after 72 h compared with strain NwIB-BC. Nevertheless, these strains did not increase the production of 9-OHAD from the conversion of phytosterols as expected, and the titer of 9-OHAD in strain NF-NfSMO was enhanced by only 9.4% (Fig. 5b). The cell growth of NF-NfSMO was significantly reduced by 20.1% compared with control strain NwIB-I-BC (Fig. 5b), which obviously compromised the conversion of phytosterols to 9-OHAD. Further, the investigation indicated that the reduced cell growth might be attributed to the dramatic increase in intracellular H_2_O_2_ level in strain NF-NfSMO compared with the strain NwIB-I-BC due to the oxidation regeneration of FAD from FADH_2_.

### Reducing H_2_O_2_ toxicity by augmenting the expression of catalase

**Fig. 6:**
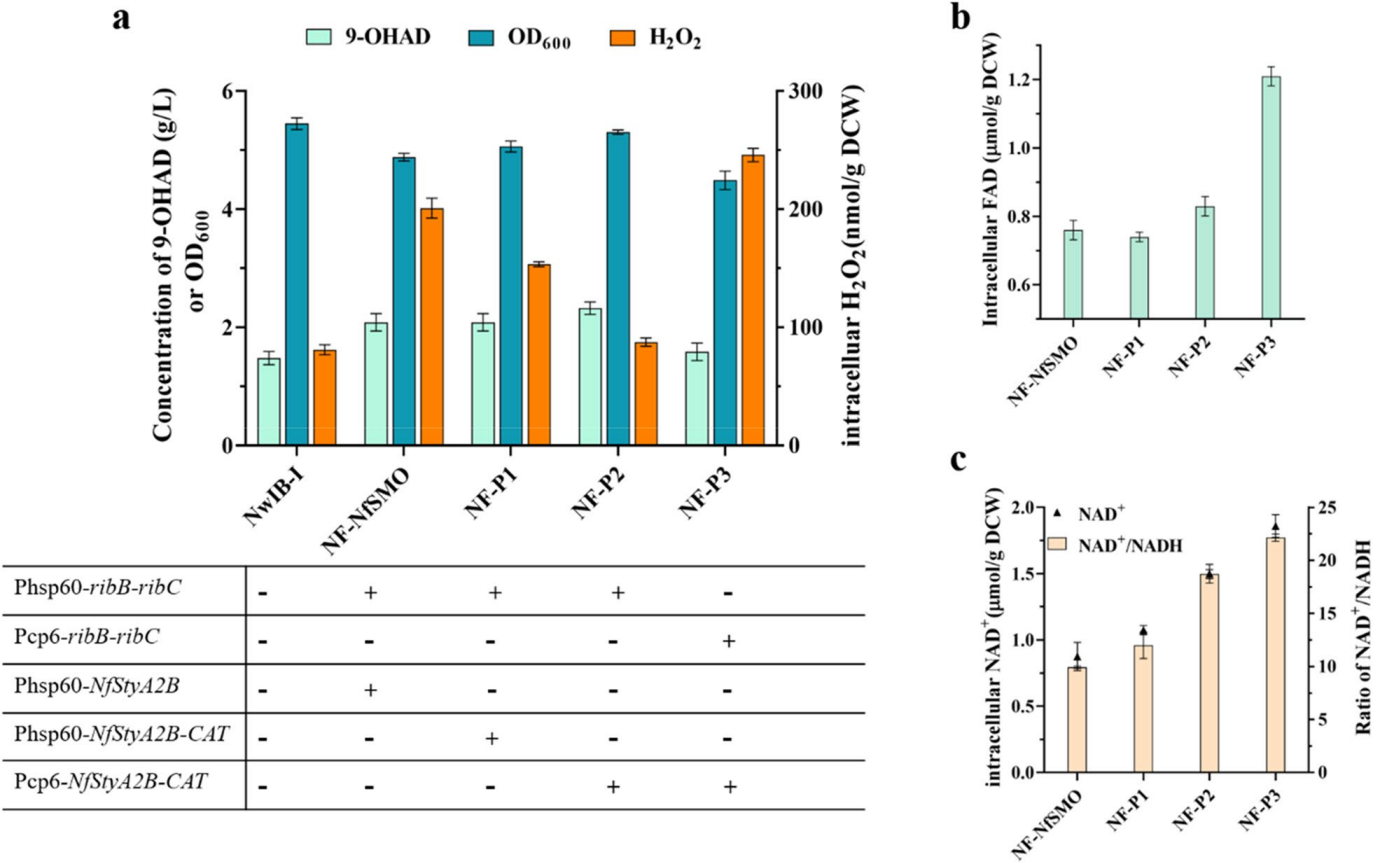
Co-expression of key genes *ribB*, *ribC*, *NfStyA2B*, and *CAT* by different promoter combinations. **a** 9-OHAD product and cell growth. **b** Intracellular FAD level, **c** NAD^+^ level, and ratio of NADH/NAD^+^ in engineered strains with combinations of different promoters and genes. All engineered strains were cultivated in fermentation medium containing 4 g/L phytosterols at 30 °C for 144 h. The data represent the mean ± standard deviation of three measurements

As H_2_O_2_ was generated from the regeneration of FADH_2_ into FAD attenuated the growth of engineered strains, the accumulation of high H_2_O_2_ concentration has the potential to damage diverse cellular components and further lead to toxic effects on cell growth [9]. This outcome was possibly on account of the lack of catalase activity for engineered strains, resulting in an inability to eliminate H_2_O_2_ in a timely fashion and allowing for an easy accumulation to a high concentration [29]. Catalase (hydrogen-peroxide oxidoreductase; EC:1.11.1.6) plays a key role in quenching and reducing the over-generated H_2_O_2_ in cells, because it because it prevents the formation of highly reactive oxygen species (ROS) by catalyzing the dismutation of H_2_O_2_ into H_2_O and O_2_ [9, 30]. It was conceivable that overexpression of CAT would be conducive to improving the conversion of phytosterols into 9-OHAD. Therefore, the CAT of *Mycolicibacterium neoaurum* was selected for overexpression in a tandem polycistronic form with the gene *NfStyA2B* in strain NF-NfSMO to efficiently achieve the CAT expression (Fig. 6a). At first, the promoter hsp60 was employed to express the polycistron, generating strain NF-P1. It showed that the intracellular H_2_O_2_ level of strain NF-P1 declined by 25.2% at 72 h compared with that of NF-NfSMO, but it was still 77.9% higher than that of strain NwIB-I. Also, the cell biomass and the 9-OHAD production of NF-P1 were only slightly higher than those of NF-NfSMO, suggesting that the CAT expression was not enough to relieve H_2_O_2_ toxicity in strain NF-P1. Therefore, the expression promoter of CAT (Phsp60) in strain NF-P1 was further replaced by a stronger promotor cp6 [31], obtaining strain NF-P2 (Fig. 6a). As expected, the intracellular H_2_O_2_ level of strain NF-P2 was greatly reduced to 34.8% of that of strain NF-NfSMO, which was close to the H_2_O_2_ level in the origin strain NwIB-I (Fig. 6a). Simultaneously, the production titer of 9-OHAD was significantly enhanced from 2.08 to 2.33 g/L by 12.0% and the biomass was also increased by 12.7% (Fig. 6a). Further, the tandem expression of *CAT* and *NfStyA2B* with promoter cp6 also resulted in 83.1% increase of NAD^+^/NADH (Fig. 6c) in strain NF-P2 compared with strain NF-NfSMO, which might be one of the reasons contributing to the enhancement of 9-OHAD production. Anyway, these results confirmed that reducing the level of over-generated H_2_O_2_ in engineered strains with enhanced synthesis and regeneration of FAD was necessary to further improve the conversion of phytosterols.

Given that the promoter cp6 performed better than Phsp60 in the overexpression of CAT, Pcp6 was used to replace Phsp60 to further enhance the expression level of *ribB* and *ribC* in strain NF-P2 to test whether the conversion of phytosterols into 9-OHAD could be further increased, resulting in strain NF-P3. The production titer of 9-OHAD and biomass of NF-P3 sharply reduced compared with that of strain NF-P2. Instead, the intracellular H_2_O_2_ level was significantly higher (Fig. 6a), although the intracellular level of FAD greatly increased from 0.82 to 1.21 μmol/g DCW (Fig. 6b). These results were similar to those of exogenously adding FMN, confirming that the high intracellular content of FAD had an obvious impact on the growth and metabolism of cells. Thus, strain NF-P2 was selected as an optimal strain for further investigation.

### Evaluating the application potential of FAD engineering strategy

**Fig. 7:**
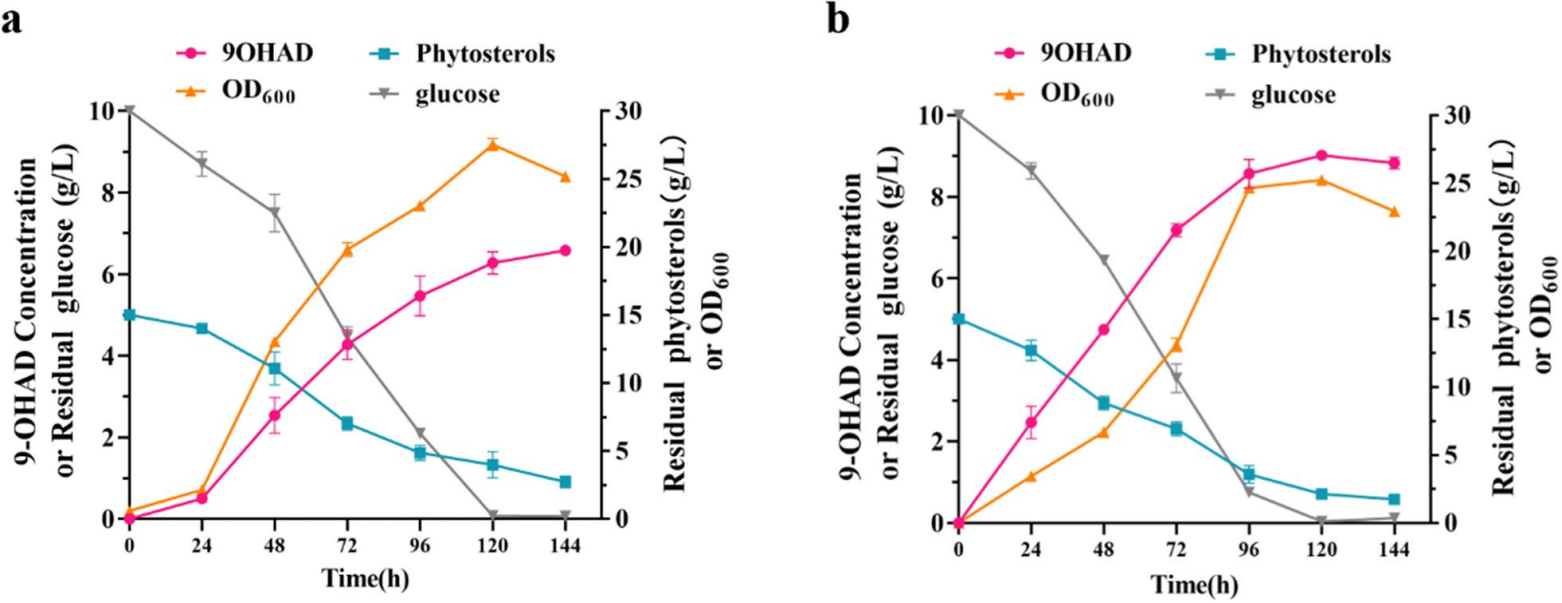
Time course of the conversion of phytosterols to 9-OHAD by **a** NwIB-I and **b** NF-P2 strain with 15 g/L phytosterols in 5.0 L fermenter. Data represent mean ± standard deviation of three measurements

The batch fermentation performances of the engineered strain NF-P2 and the original strain NwIB-I were compared in a 5-L bioreactor after adding 15 g/L phytosterols to evaluate the effect of FAD engineering strategy on the conversion of phytosterols into 9-OHAD in the industry. Strain NwIB-I showed fast growth from 24 to 120 h, and the maximum OD_600_ reached 27.5 after 120 h and the concentration of 9-OHAD reached 6.35 g/L after 144 h (Fig. 7a). In contrast, the growth period of strain NF-P2 was reduced in 24 h, and its biomass (OD_600_ = 25.3) peaked after 96 h. Despite an 8.2% decrease in maximum biomass, the maximum 9-OHAD production titer of NF-P2 increased by 42.1%, reaching 9.02 g/L after 120 h (Fig. 7b). Hence, the 9-OHAD time–space productivity of strain NF-P2 reached 0.075 g/(L h), which was 66.7% higher than that of NwIB-I (0.045 g/L/h). Further, compared with the previously reported 9-OHAD producers with vegetative cell fermentation, NF-P2 achieved the highest molar yield of 9-OHAD (Table 1). These results demonstrated that augmenting the supply and regeneration of FAD was a considerably effective strategy to boost the conversion of phytosterols into steroid synthons.

**Table 1:** Comparison of microbial 9-OHAD producers using phytosterols

Strains	Substrates (g/L)	Yield (g/L)	Molar yield (%)	References
NwIB-yV	15	7.33	67.0	[3]
*M. neoaurum* MP1	13	5.72	61.0	[31]
HGMS2^Δkstd211 + Δksh226^	80	42.4	72.9	[35]
NF-P2	15	9.02	80.6	This study

## Discussion

The conversion of cheap phytosterols into steroid synthons is one of the most important steps in producing diverse value-added steroid hormones in the pharmaceutical industry [1, 32]. These steroid synthons, such as AD and 9-OHAD, are generally obtained via the side-chain oxidative degradation of phytosterols by *Mycolicibacterium* species, and this catabolism is accompanied by the generation of a large number of reduced cofactors NADH and FADH_2_ (Fig. 1). The cofactor regeneration engineering of NAD^+^ has been demonstrated to be an effective way to enhance the productivity of steroid synthons [12, 14, 20]. Nevertheless, the specific effects of the supply and regeneration of FAD on the biotransformation of phytosterols into steroid synthons have not been investigated earlier. In this study, we substantiated that the supply of FAD played an essential role in the catabolism of phytosterols, and augmenting the synthesis and regeneration of FAD was an effective strategy to improve the conversion of phytosterols into 9-OHAD in mycolicibacterial strains.

The catabolism of sterols in *Mycolicibacterium* species is an induction process. Hence, the key enzymes involving in the catabolic pathway of phytosterols are only expressed in the presence of sterols. Obviously, mycolicibacterial strains require a large supply of FAD to drive sterol transformation in the presence of sterols compared with the absence of sterols (Fig. 2a). Sterols in natural environment are not a rich substance, and not a preferred carbon source for *Mycolicibacterium* to use. However, the added concentration of sterols is much higher under industrial conditions than in the natural environment. In this case, the insufficient supply of FAD can become a key factor limiting the transformation of sterols due to the large demand for FAD in the catabolism of sterols, which was confirmed in this study by augmenting the biosynthesis of FAD in the 9-OHAD-producing strain NwIB-I (Fig. 3b). Among the six key genes involved in the biosynthesis of FAD, *ribB* and *ribC* showed to be the rate-limiting genes for FAD biosynthesis in *Mycolicibacteria* and the individual augmentation or co-augmentation of the two genes could obviously increase intracellular FAD level (Fig. 3c) and the conversion of phytosterols into 9-OHAD (Fig. 3d). This result was similar to that obtained in the *S. cerevisiae*, in which enhancing the synthesis of FAD by combinatorically expressing *Bacillus subtilis BsRIBBA* and *Thermotoga maritima TmBiFADS* could significantly drive the biosynthesis of phenolic acid [33]. In additional, the results might be further explained in terms of gene expression levels, similar to the study of Manan et al., in which in which *RIB3* and *FAD1* (correspond to *ribB* and *ribC*, respectively) expression significantly increased during flavins production phase compared to growth phase [26]. Therefore, it is hypothesized that *ribB* and *ribC* might be expressed at higher levels than other pathway genes in the FAD biosynthesis in *Mycolicibacterium.*

Since the mass formation of reduced cofactor NADH and FADH_2_ is a distinct feature of the catabolism of phytosterols in *Mycolicibacterium*, the ratio of NADH/NAD^+^, as well as FADH_2_/FAD, is much higher in the presence of sterols than in the absence of sterols, attenuating the active conversion of sterols [16]. It was confirmed that reducing the ratio of NADH/NAD^+^ could validly enhance the conversion of sterols into different steroid synthons [12, 14, 20], implying that reducing the ratio of FADH_2_/FAD might also be an effective way to boost the conversion of sterols into steroid synthons. Unfortunately, no accurate method was available to detect intracellular FADH_2_, especially for sterol conversion in *Mycobacterium*, since free O_2_ rapidly reacted with the unstable FADH_2_ to produce H_2_O_2_, superoxide, and hydroxyl radical [34]. Although the ratio of FADH_2_/FAD could not be measured, it was confirmed that the elevation of FAD concentration, as well as the recycling of NAD^+^ from NADH by introducing a heterologous NADH-FAD(H_2_)-dependent styrene monooxygenase NfSMO from *N. farcinica*, could conspicuously boost the transformation of phytosterols into 9-OHAD (Fig. 5b), suggesting that the ratio of FADH_2_/FAD could have been reduced. This hypothesis was further proved based on the fact that the intracellular level of H_2_O_2_ after the expression of gene *NfStyA2B* had a striking improvement of 104.5% because H_2_O_2_ was the product of the regeneration of FAD from FADH_2_ (Fig. 4d). On the one hand, this result could be attributed to the increase in the oxidative regeneration of FAD from FADH_2_ due to the enhancement of FAD concentration. On the other hand, as a class of a new class of NADH- and flavin-dependent single-component flavoprotein monooxygenases, similar to a fusion protein, NfSMO contains components of oxygenase and FAD oxidoreductase [28]. In the absence of a suitable substrate such as styrene, the electron acceptor oxygen acts as the substrate, accepting all the hydrogen protons from FADH_2_ to produce the peroxide. In the presence of suitable substrates, the FADH_2_ regeneration rate of FAD will be greater than the autoxidation process of FADH_2_, accompanied by the generation of H_2_O instead of H_2_O_2_ [35]. However, overexpression of NfSMO leads to a significant increase in intracellular H_2_O_2_ level, which inferred that the substrate for NfSMO might be oxygen. The possibility of other metabolites cannot be ruled out, but most likely not sterol intermediates, as other suspected steroid by-products were not detected by HPLC (date not shown). Further analysis of metabolomics may be a better alternative to know what substrates NfSMO acts on. Certainly, the increased production of H_2_O_2_ seriously inhibited the growth of the engineered strain NF-NfSMO (Fig. 5b). Therefore, an H_2_O_2_-neutralizing strategy was used to quench the overproduced H_2_O_2_ to reduce the toxic effect on cell growth. Further, the over-generated H_2_O_2_ was successfully neutralized by overexpressing the endogenous *CAT*, and the conversion of phytosterols into 9-OHAD was further enhanced after strengthening the supply and regeneration of FAD due to the restoration of cell growth.

Although promoting the supply and recycling of FAD has been demonstrated to be conducive to the bioconversion of phytosterols to steroid synthons, excessive FAD supply is not always beneficial. The results of exogenously adding FMN into the conversion of phytosterols indicated that adding 0.4 mmol/L FMN resulted in an optimal intracellular FAD concentration, which was about 0.64 μmol/g DCW. However, even higher FAD was not conducive to the biotransformation of phytosterols. Similarly, overexpressing the rate-limiting enzyme RibB and RibC of the FAD biosynthesis pathway could also significantly enhancing the intracellular FAD concentration, an optimal FAD concentration, 0.62 μmol/g DCW, was achieved by augmenting the expression of *ribB* and *ribC* under the control of Phsp60, the effect of which was similar to that of exogenously adding 0.4 mmol/L FMN. Along with the increase in the intracellular concentration of FAD, cell growth was significantly inhibited, demonstrating that the high concentration of FAD caused obvious toxicity to cells, which was attributed to the H_2_O_2_ generated from the regeneration of FAD in the cells. Thus, augmenting the cyclic regeneration of FAD by introducing styrene monooxygenase NfSMO in strain NwIB-I-BC further decreased the cell biomass due to the enhanced FAD concentration. In this study, an optimized *CAT* expression was used to neutralize the over-generated H_2_O_2_, and the resulting strain NF-P2 achieved a 66.7% increase in the time–space productivity of 9-OHAD compared with the original strain NwIB-I, substantiating the importance to balance the relationship between intracellular FAD content and the H_2_O_2_ level. Based on strain NF-P2, further augmentation of FAD synthesis by replacing the expression promoter hsp60 of *ribB* and *ribC* with a stronger promoter cp6 led to a significant decrease in 9-OHAD production and cell growth due to the sharp increase in the H_2_O_2_ level. These results implied that the over-generated H_2_O_2_ resulted in irreversible effects on the normal growth of cells and the conversion activity of phytosterols if it could not be neutralized in time. In this case, further augmentation of the *CAT* expression may not be the only means, and the biosynthesis of the unique antioxidants, including ergothioneine and mycothiol in *Mycolicibacterium* for quenching reactive oxygen species may be a complementary strategy to neutralize the overproduced H_2_O_2_ in the conversion process of phytosterols to steroidal synthons [9].

## Conclusions

In this study, we have substantiated the essential role of FAD in the conversion of phytosterols to steroid synthons and proposed a FAD engineering strategy to effectively boost the conversion of phytosterols to steroidal synthons by optimizing the supply and recycling of FAD and meanwhile neutralizing the over-generated H_2_O_2_ from the regeneration of FAD. Finally, we achieved a high 9-OHAD-producing strain, NF-P2, with the highest molar yield of 9-OHAD among the reported strains (Table 1). These results suggested that the cofactor engineering, including the supply and recycling of FAD and NAD^+^, should be adopted as a parallel strategy with pathway engineering to improve the productivity of the industrial strains in the conversion of phytosterols to steroid synthons.

## Materials and methods

### Strains and plasmids

The strains and plasmids and used in this study are listed in Table S1. The engineered *M. neoaurum* strains were all derived from the wild-type *M. neoaurum* ATCC 25795. The typical 9-OHAD-producing strain NWIB-I was constructed by deleting *kstD1*, *kstD2*, and *kstD3* in *M. neoaurum* ATCC25795 [3]. *Escherichia coli* DH5α was used for plasmid construction.

### Reagents, media and culture conditions

KOD-Multi and EpiDNA polymerase (Toyobo Biotech. Co., Ltd., Shanghai, China) and PrimeSTAR Max (Takara Bio. Inc., Shiga, Japan) were used to perform the polymerase chain reaction (PCR). Hieff Clone Plus Multi One Step Cloning Kit [Yeasen Biotechnology (Shanghai) Co., Ltd., Shanghai, China] and T4 ligase (Thermo Fisher Scientific Inc., MA, USA) were employed to assemble nucleotide sequences. FMN was obtained from Shanghai Macklin Biochemical Technology Co., Ltd. (Shanghai, China). A mixture of phytosterols was purchased from Shaanxi Sciphar Natural Products Co., Ltd. (Shaanxi, China). The substrate of phytosterols (100.0 g/L) was emulsified in Tween 80 (5% *w*/*v*) aqueous solution at 121 °C for 30 min before use. Other reagents were prepared as previously reported [11].

*E. coli* DH5α was cultured in 5 mL of Luria–Bertani (LB) medium (10.0 g/L tryptone, 5.0 g/L yeast extract, 10 g/L NaCl, and pH 7.0) for the growth at 37 °C and 220 rpm for 12 h. *M. neoaurum* strains were first grown in 5 mL of LB medium at 30 °C for 48 h. Then, the cell suspensions (3 mL) were inoculated into 30 mL of seed medium (20.0 g/L glycerol, 2.0 g/L citric acid, 0.05 g/L ammonium ferric citrate, 0.5 g/L K_2_HPO_4_, 0.5 g/L MgSO_4_·7H_2_O, 1.65 g/L KNO_3_, and pH 7.5) for 48 h (OD_600_ = 3.5–4.0).

For the biotransformation in growing cells, 3 mL of the cultivated cells were transferred to 30 mL of fermentation medium (10.0 g/L glucose, 2.0 g/L citric acid, 0.05 g/L ferric ammonium citrate, 0.5 g/L MgSO_4_·7H_2_O, 0.5 g/L K_2_HPO_4_, 2.0 g/L KNO_3_, and pH 7.5) with 4 g/L phytosterols. All the shake flask experiments were performed using 4 g/L phytosterol at 30 °C under aerobic conditions. The number of colony-forming units per milliliter of the medium was used to assess the growth phenotype of cells.

For the batch fermentations, 15 mL of the cultivated seed cell suspension was transferred to 150 mL of the second-stage seed medium. After 48 h of cultivation (OD_600_ = 5.0), 450 mL of the second-order seed culture was transferred to a 5-L fermenter (blbio-5GJ-3GJ-5; Bailun Bio-Technology Co., Ltd., Shanghai, China) containing 3 L of the transformation medium. Meanwhile, a mixture of phytosterols (100.0 g/L) was emulsified with hydroxypropyl-β-cyclodextrin (HP-β-CD; RSC Chemical Industries Co., Ltd., Jiangsu, China) (0.25 *w*/*w*) at 121 °C for 60 min. Then, the emulsified phytosterols were added to the fermentation medium, and the final concentration was 15 g/L. The following fermentation was carried out at 30 °C with airflow at 1.0 volumes of air per volume of medium per minute (vvm).

### Construction of recombinant strains

All primers were synthesized by Tsingke Biotechnology Co., Ltd. (China) and are listed in Table S2. One-component styrene monooxygenase genes from *Rhodococcus opacus* (*RoStyA2B*), *Paenarthrobacter aurescens* (*PaStyA2B*), and *N. farcinica* (*NfStyA2B*) were codon-optimized for expression in actinomyces and synthesized by Tsingke Biotechnology Co., Ltd. (China) (Additional file 1: Sequences).

The genetic sequence of the cp6 promoter was obtained from a previous study [31]. Further, cp1, cp2, and cp3 fragments from pMV261 plasmids were amplified using primer pairs cp-F/R1, cp-F/R2, and cp-F/R3, respectively, and fused to cp6 fragment by primers cp6-F and cp6-Fusion through overlap extension PCR. The fusion fragments were digested with *Kpn*I and *Bam*HI and then ligated to the *Kpn*I and *Bam*HI sites of the pMV261 plasmid by T4 ligase to obtain the plasmid pMV261*. The gene overexpression in *M*. *neoaurum* strains was performed with plasmid pMV261 and pMV261* as previously described [9]. The cassettes P_hsp60_/P_cp6_ − ribB − ribC and P_hsp60_/P_cp6_ − NfStyA2B − CAT were amplified using the primers 2612-F/R and ligated to the *Xba*I site of the plasmid pMV261 or pMV261*. All the constructed plasmids were transferred to mycobacterial cells with electroporation.

### Measurement of intracellular FAD and H_2_O_2_ concentrations

The intracellular FAD concentration (excluding FADH_2_), was measured with an FAD assay kit (BioVision, Inc., CA, USA). Briefly, the washed cells were homogenized in FAD assay buffer, and the suspensions were centrifuged at 12,000 g for 5 min to remove the insoluble material. After that, the deproteinization sample preparation kit (BioVision Inc., CA, USA) was used to release FAD. The reaction mixture contained 46 mL of FAD assay buffer, 2 mL of fluorescent peroxidase substrate, and 2 mL of FAD enzyme mix. FAD could be detected by either colorimetric (OD = 570 nm) or fluorometric (Ex/Em = 535/587 nm) methods. H_2_O_2_ was quantified using a hydrogen peroxide content assay kit (Beijing Solarbio Science & Technology Co., Ltd., Beijing, China). The extraction and quantification of H_2_O_2_ were performed following the manufacturer’s protocols.

### Determination of NAD^+^ and NADH levels

The intracellular levels of NAD^+^ and NADH were measured according to the operating manual of the NAD^+^/NADH Assay Kit WST-8 (Beyotime, Shanghai Co., Ltd., Shanghai, China). The mycobacterial cultures were collected by centrifugation. The cells were washed twice with phosphate-buffered saline, and 1 OD of cells was resuspended in NAD^+^/NADH extraction buffer. The extraction and quantification of NADH and NAD^+^ were performed following the manufacturer’s protocols.

### Sterol transformation and analysis

The bioconversion of phytosterols was detected as previously described [11]. The target product 9-OHAD was analyzed by high-performance liquid chromatography (Agilent Technologies, CA, USA) with an Agilent reversed-phase C18 column (250 × 4.6 mm^2^; 30 °C) using methanol/water (80:20, *v*/*v*) as the mobile phase at a flow rate of 1 mL/min with ultraviolet detection at 254 nm. The amounts of phytosterols were determined using a gas chromatography system 7820A (Agilent Technologies, CA, USA) with an Agilent DB-5 column [30 m × 0.25 mm (i.d.) × 0.25-μm film thickness]. The oven temperature was controlled as follows: 200 °C for 2 min, 200–280 °C within 4 min, 280 °C for 2 min, 280–305 °C within 1.5 min, and 305 °C for 10 min. The inlet and flame-ionization detector temperatures were maintained at 320 °C. The nitrogen carrier gas flow was 2 mL/min at 50 °C.

## Supplementary Information

**Additional file 1. Table S1**: Plasmids and Strains used in this study. **Table S2**: Primers used in this study. Sequences. codon-optimized genes *RoStyA2B*, *PaStyA2B*, *NfStyA2B* in this study.

## Data Availability

All data generated or analyzed during this study are included in this published article and its additional information fles.
