# Supplementary material for: Driving the conversion of phytosterol to 9α-hydroxy-4-androstene-3,17-dione in Mycolicibacterium neoaurum by engineering the supply and regeneration of flavin adenine dinucleotide
Source: Biotechnol Biofuels Bioprod. 2023 Jun 8;16:98. doi: 10.1186/s13068-023-02331-1 (PMC10251532; doi:10.1186/s13068-023-02331-1)
Supplement: Supplementary file 1 — Additional file 1. Table S1: Plasmids and Strains used in this study. Table S2: Primers used in this study. Sequences. codon-optimized genes RoStyA2B, PaStyA2B, NfStyA2B in this study. [file 13068_2023_2331_MOESM1_ESM.docx]

**Driving the conversion of phytosterols to 9α-hydroxy-4-androstene-3,17-dione in *Mycolicibacterium neoaurum* by engineering the supply and regeneration of flavin adenine dinucleotide**

Lu Song^1,3^, Jie Ke^1,3^, Zhi-Kun Luo^1,3^, Liang-Bin Xiong^1,2^, Yu-Guo Dong^1*^, Dong-Zhi Wei^1,3^, Feng-Qing Wang^1,3*^

^1^ State Key Laboratory of Bioreactor Engineering, Newworld Institute of Biotechnology, East China University of Science and Technology, Shanghai 200237, China.

^2^ Jiading District Central Hospital Affiliated Shanghai University of Medicine and Health Sciences, Shanghai 201800, China.

^3^ Key Laboratory of Biocatalysis and Intelligent Manufacturing (ECUST), China National Light Industry, Shanghai 200237, China.

# Corresponding author

* E-mail: dongyuguo@ecust.edu.cn, Tel: +86-21-6425-3287. Fax: +86-21-6425-0068.

* E-mail: fqwang@ecust.edu.cn, Tel: +86-21-6425-3287. Fax: +86-21-6425-0068. ORCID: 0000-0002-3473-5991.

**Supplementary Table 1**

**Table 1** Plasmids and Strains used in this study

| **Name** | **Description** | | **Source** | |
| --- | --- | --- | --- | --- |
| Plasmids |  | |  | |
| p261 | Shuttle vector of *M. neoaurum* and *E.coli*, carrying the heat shock (hsp60) promoter, *Kan^R^* | | [1] | |
| p261* | Shuttle vector of *M. neoaurum* and *E.coli*, carrying artificial promoter CP6, *Kan^R^* | | This work | |
| pFAD-A | p261，P_hsp60_-*ribA* | | This work | |
| pFAD -B | p261，P_hsp60_-*ribB* | | This work | |
| pFAD -C | p261，P_hsp60_-*ribC* | | This work | |
| pFAD -D | p261，P_hsp60_-*ribD* | | This work | |
| pFAD -F | p261，P_hsp60_-*ribF* | | This work | |
| pFAD -H | p261，P_hsp60_-*ribH* | | This work | |
| pFAD -BC | p261，P_hsp60_-*ribB-ribC* | | This work | |
| pFAD-RoSMO | p261，P_hsp60_-*ribB-ribC-* P_hsp60_-*RoStyA2B* | | This work | |
| pFAD-PaSMO | p261，P_hsp60_-*ribB-ribC-* P_hsp60_-*PaStyA2B* | | This work | |
| pFAD-NfSMO | p261，P_hsp60_-*ribB-ribC-* P_hsp60_-*NfStyA2B* | | This work | |
| pFAD-No-Cat | p261，P_hsp60_- *NfStyA2B -CAT* | | This work | |
| pFAD* -BC | P261*，P_cp6_-*ribB-ribC* | | This work | |
| pFAD*-No-Cat | P261*，P_cp6_- *NfStyA2B -CAT* | | This work | |
| pNF-1 | p261, P_hsp60_-*ribB-ribC-* P_hsp60_- *NfStyA2B -CAT* | | This work | |
| pNF-2 | p261- P261*, P_hsp60_-*ribB-ribC-* P _cp6_- *NfStyA2B -CAT* | | This work | |
| pNF-3 | P261*，P_cp6_-*ribB-ribC-* P_cp6_- *NfStyA2B -CAT* | | This work | |
| Strains |  | |  | |
| *E. coli* DH5α | | *E. coli* strain for cloning | Tian Gen Co., Ltd. |  |
| *M.neoaurum* ATCC 25795（Mn） | | Wild type strain, the starting strain | ATCC |  |
| NWIB-I | | Deletion of *kstD*, *kstD2*, *kstD3* in *M. neoaurum* ATCC 25795, 9-OHAD-producing strain | [2] |  |
| NWIB-I-A | | NWIB-I containing p261-A | This study |  |
| NWIB-I-B | | NWIB-I containing p261-B | This study |  |
| NWIB-I-C | | NWIB-I containing p261-C | This study |  |
| NWIB-I-D | | NWIB-I containing p261-D | This study |  |
| NWIB-I-F | | NWIB-I containing p261-F | This study |  |
| NWIB-I-H | | NWIB-I containing p261-H | This study |  |
| NWIB-I-BC | | NWIB-I containing pFAD-BC | This study |  |
| NWIB-I* | | NWIB-I containing p261 | This study |  |
| NF-RoSMO | | NWIB-I containing pFAD-RoSMO | This study |  |
| NF-PaSMO | | NWIB-I containing pFAD-PaSMO | This study |  |
| NF-NfSMO | | NWIB-I containing pFAD-NfFMO | This study |  |
| NF-P1 | | NWIB-I containing pNF-P1 | This study |  |
| NF-P2 | | NWIB-I containing pNF-P2 | This study |  |
| NF-P3 | | NWIB-I containing pNF-P3 | This study |  |

**Supplementary Table 2** Primers used in this study

| **Primers** | **Description** |
| --- | --- |
| 261-ribA-F&R | CGCGGATCCATGACACGGCTGGATTCCGTCGAAC& |
|  | CCCAAGCTTTCACAGCGCGCCACCGGGTTG |
| 261-ribB-F&R | CGCGGATCCATGAGAACGACGAGCCAGTCGG& |
|  | CCCAAGCTTGAACGTTCTCGAACAGGCCTATTGA |
| 261-ribC-F&R | CGCGGATCCATGTTCACCGGAATCGTGGAAGAG& |
|  | CCCAAGCTTCTACTCGGCGAACGTGACCGCTGA |
| 261-ribD-F&R | CCGGAATTCATGCGGCTGGCGATCGAGCAGTC& |
|  | CCCAAGCTTGTGCGCCTGAGCCTGGTACCCAATTGA |
| 261-ribF-F&R | CCGGAATTCGTGCAGCGGTGGCGGGGCC& |
|  | CCCAAGCTTGCGCTCGATTCTCGCTGCGGGCTG |
| 261-ribH-F&R | CGCGGATCCGTGGACGCCTCCACGGTGTCGC& |
|  | CCCAAGCTTTCATGAGGCCAGGTCGCGCAGC |
| rbs-ribC-F&R | GAACAGGCCTATTGAACGCGTAAGAGGAGAAATGTTCACCGGAATCGTGGAAG& |
|  | CGTACGCTAGTTAACAAGCTTTCAGCGGTCACGTTCGCCGAGT |
| cp6-BC- F&R | TTGACAAAAAAATCAAAATATGGTATAATGGTTGACAATTAATCATCGGCTCGTATAATGCCCCGATCCGGAGGAATCAC& |
|  | CTGCGCCCGGCCAGCGTAAGTAGCGGGGTTGACAAAAAAATCAAAATATG |
| cp6-rbs-cat-F&R | CTCGGCGAACGTGACCGCTGAAAGCTTAAGAGGAGAAATGTTCACCCACAACAA& |
|  | CTGGCAGTCGATCGTACGCTAGTTAACTCAGCTCGTGGCGGCGTGCTG |
| 261-Rosmo- F&R | CAATTGCGGATCCAGCTGCAGAATTCATGCAGTGGCACGAGAAGATC& |
|  | TTAACTACGTCGACATCGATAAGCTTATTCTGGGCGTCCGCCAGG |
| 261-Pasmo- F&R | CAATTGCGGATCCAGCTGCAGAATTCATGCGGAAGATCGCCATCGTG& |
|  | TTAACTACGTCGACATCGATAAGCTTGAAGTCCGGGTGGGACGCGGTG |
| 261-Nfsmo- F&R | CAATTGCGGATCCAGCTGCAGAATTCATGTCCGCCCAGCGCCGGATCG& |
|  | GATCGCCGCCGACCATCCGGACTTCGCCTGAAAGCTTATCGATGTCGACGT |
| 261-Nf-cat- F&R | CCGACCATCCGGACTTCGCCTGAAAGCTTAAGAGGAGAAATGTTCACCCACAACAA& |
|  | TTAACTACGTCGACATCGATAAGCTTTCAGCTCGTGGCGGCGTGCTGC |
| cp6-F | GGTACCAGATCTTTAAATCTAGAGGTGACCACAACGAC |
| cp6-R1 | **tgtcaaccattataccatattttgatttttttgtcaa**CCCCGCTACTTACGCTGGC |
| cp6-R2 | CGGGG**cattatacgagccgatgattaattgtcaaccattatacc** |
| cp6-R3 | CCATTGCGAAGTGATTCCTCCGGATCGGGG**cattatacgag**  GGATCCGCAATTGTCTTGGCCATTGCGAAGTGATTCC |
| cp6-infusion-R |  |
| 2612-F&R | TGGCATCCGTGGCGCGGCCGCGGTACCGGTGACCACAACGACG& |
|  | CACCTCTAGATTTAAAGATCTGGTACCTAAAACGAAAGGCCCAGTCTTTCGAC |

**Notes**: The restriction enzyme sites were underlined，the sequences of cp6 promotor were showed in bold and black little letters

**Sequences：**

*RoStyA2B* ATGCAGTGGCACGAGAAGATCCTGCTGACGGTGGTCGTAGCCCGGACGGACGACTCGGGCACCGCGCGGACCGAGCAGCGGGAGGAGCCGACCGTCCGGGACCCGCGTTACGACATACTCTTCGAGCCGGTCGAGATCGGTCCGGTGACCGCGCGTAACCGGTTCTTCCAGGTGCCGCACTGCAACGGCATGGGCTACCGCGACCCGTCGGGGGAGGCCTACATGCGCCGCGTGAAGGCCGAAGGGGGCTGGGCGGTGGTGTGTACCGAGCAAGTCGAGATCCACCCCACCTCGGACATCGGGCCTTTCATCGAGCTGCGGCTGTGGGACGATCAGGACCTGCCGGCCCTGACCCGGATCTCCGAGAAGATCCACGAGGGCGGCAGCCTGGCCGGGATCGAGCTGGCCCACAACGGTCTCAACAGCCCGAACCTCATCAGCCGGGAAACCCCGCTCGGCCCGCAGAACCTCCCCGTCGTCTCCTGGAACTACGACCCCGTGCAGGCCCGCGCCATGACCAAGTCCGACATCGCCGACCTCCGGCATTGGCACAAGGAGGCGGTGCGGCGCTCGCTCAAGGCGGAGTACGACATCGTCTACGTGTACGCGGGGCACGCCATCGGGGGACTGCACCACTTCCTCTCCAGACGGTACAACAACCGGAGCGACGAGTACGGCGGATCGCTCACCAACCGCGTCCGCCTCCTGCGCGAGATCCTCGAAGACTCCCGCGAGGTCGCCGACGGCAAAGCCGCCGTCGCCTGCAGGATCTCGGTGGACGAGCTGCTCGGGGACGAGGGCATCACCCGCAGCGAGATCGAGGACGTCATCGGCCTCCTCGGCGAGACCCCGGACCTGTGGGACTTCGTGCTGGGCTCCTGGGAGGACGACTCGGTCACCAGCCGCTTCGGCCCCGAGGCCGAGCAGGAGCCGTACGTGCGGGGTCTGAAGCAGCTGACCACGAAGCCGGTGGTGGGCGTGGGCCGCTTCACGAGCCCCGACACCATGCTGCACCAGGTCAAGAGCGGCATCCTGGACTTCATCGGAGCCGCGCGCCCCTCGATCGCCGACCCCTACCTGCCGCGGAAGATCGAGGAGGGCAACCTGGAGGACATCAGAGAGTGCATCGGCTGCAACATCTGCGTGAGTGGTGACTTCACCATGTCGCCGATCCGCTGCACCCAGAACCCGACGATGGGCGAGGAGTTCCGGCGGGGCTGGCACCCGGAGCGCATCAAGGTCCGGTCCACCGACTCCAACGTCCTGGTGGTCGGGGCGGGCCCCGCGGGCCTGGAGGCCGCCCGAGCGCTCGGCCAGCGCGGCTACCACGTGTCCCTGGCCGAAGCGTCGCGGCAGTTGGGAGGTAGGGTTACTAGGGAGAGCAAGCTGCCCGGCCTGTCCGCCTGGATCAGGGTCGTCGACTACCGGGAGCAGCAGCTCCGCAAGCTCCCCGACGTGGACGTCTTCATGGAGTCTGAGATGACGGCGGATGACGTGATCGAAAACGACTTCCACCACGTCGTGGTCGCGACCGGCAGCCACTGGCGGCGCGACGGGGTCGGCCGGTGGCACACCCACGCCGTCCCGGTCGATGAGCACGCGGACGTACTGACGCCGGACGACGTCATGGCCGGCTTGCGCCCGCGCGGCAACGAGGTGGTTCTCTTCGACGACGACCACCACTATCTCGGTGCCGTGGTGGCCGAGCTGCTGGCCAAGGAGGGCTTCGACGTGACGCTGATCACGCCCGCCGCTCATGTTTCCCAGTGGACCACCAACACCATGGAGGTGGTCCGCATCCGCAAGCGGATCATCAAGGCGGGCGTCCACGTCCAGGTCAACCGAGCAGTAACGGCCGTCACCCCCCAGGGCGTCCGCACGGTCTGCGCCTTCACCGGCGACGAGCAGTTCGTCGACGCGGACTCCGTGGTCCTGGTCACGGCGCGGCTGCCCCACGACGGCCTTTTCCACGAGCTCCAGTCCCGAGAGTCGGAGTGGGCGGACGCCGACCTGCGCTCCGTCCGTGGCATCGGCGACGCCTGGGCCCCCTCCACGATCGCGGCCGCGGTGTGGTCCGGCCACCGCTACGCCGAGGAGCTCGACGAACCGCCGGCGCCGGGCCCAGTCACCTTCCGCCGCGAGGTCACCGGACTGTCCATCGAACCGGTGGAGAAGTTCTCCGTCCCCCCCCTGGCGGACGCCCAGAAT

*PaStyA2B*

ATGCGGAAGATCGCCATCGTGGGCGCGGGTGAGAGCGGCGCGCAGCTCGCGCTCGGCCTGCAGCGCGACGGCTACGCCGTCACCCTGCTCTCGGACCGCTCCGCGGCCCAGATCCGCACCGGGAAGGTCATGAGCTCCCAGTGCATGTTCTCCACCGCGCTGGACGCGGAGGCCCACATGGGCACCGCGCTCACCGAGTTCTACGAGAGCGGCGCGGTGCCCGGTATCACGTCCATCCGGCTGCGGGTCGACTCGGACGAGCCGATCGAGTGGGAGGCTCCGCTCGACGGGCCGGCCCGCAGCATCGACCAGCGCATCAAGTCCGCCCTGTGGATCGAGACCTTCGTCGCCGCGGGTGGGGACTTCCGCATCGAAAAGGTGACGCCCCGGATGCTGGAGGAGCTGGCGCACGACTACGAGCTGGTGATCGTCTCCACCGGCAAGGGCGAGATCGGCCAGATCTTCCCGCGCGACAAGGCCAAGTCCCCCTTCGACCGTCCGCAGCGGGTGTTGGCCCTCAACTACGTGACGTCGGACGACGGCGCAAGCGCCGCCGGGACCGGAGGGGCGGGCGATGCGATCCGGATGTCGATGGCGCCCGGCGTCGGGGAGTTCTTCACCTTCCCGGGCCTAACCGTATCGGGCCCGTGCCGCATGATGGTCTTCGAGGGCGTCGTGGGCGGCCCGATGGACCGCTGGGCCGACGTGGGTACTCCCGAGGAGCAGCTGGAGCGCAGTCTCGAAATCCTCCGGGACCACTTCCCCCACGAGGCCGAGAACTTCGCCGCCGCCGAGCTGACGGACAGCGGCGCCACGCTGCTCGGTCGGATCACCCCGACCGTGCGTTTCGCCGTGGGGGAACTGGGCAACGGCAAGCTCGTCTTCGGCCTGGGTGACGCCGTCGTGCTGAACGACCCCCTCACCGGCCAGGGCTCGAACAACGCCACCCTGGCCGCCAAATACTACCTGGACAGCATCATCCGCCGGGGCCAACAGCCGTTCGACCGGGCCTGGATGGAGCGCACCTTCGACGAGTTCTGGCGGGGCTGGGGCCAGTGGGCGGTCGAGTGGACGAACGACCTGCTGAAGCCGCGCAGGGACCACCAGAAGACGTTACTGCGCGAGGCGGCAGACCACCCCGCGCTCGCGGCCTCCATCGTCTCGGGCTTCGACGACCCCCGGACCTCGTACCCCTGGTGGTTCGATCCGACGGCGGCGGCCGACTTCATGCAGGCCCGCAAGGAGGACGACGCGGCCGCCTTCGACGTCCGAGACTTCCGCGGAGCGCTCGGCCAGTTCGCCACCGGGGTGACGGTCGTCACCACCGTCTCGGCCGACGGGCGCAAGGTCGGGATGACCGCCAACTCCTTCACATCGGTGTCCATGGACCCGCCCCTGGTGCTGTGGTGCCCGTCCAAGCGGGCGCCGTCCCTGGGCGACTTCGAGGACGCCACGCACTTCGCCATCAACATTCTGGCGAGCGACCAGCACGTCCTCTCTCGGCAGTTCGCAACCCCGGCCACCGACAAGTTCGCGGGGGTGGAAACGGCGGACGGCATCGCCGGAGTTCCCCTCCTGGAGGGCGCGGTAGCCACGTTCCAGTGCCGGACCGTGGCGCGGCACGACGCCGGCGACCATGTCATCTACGTCGGCGAAGTCGAGTCCTACAACCACCAGGACGGAGCTCCCCTGGTGTTCCACGGCGGCAAGTACCACGCCACCGCGTCCCACCCGGACTTC

*NfStyA2B* ATGTCCGCCCAGCGCCGGATCGCGATCGTCGGCGCCGGCCAAGCGGGGGCGCTCGTCGCGCTGGGCCTGCGGCGGCACGGCTGCGCCGTCACCTTAATAGGCGACCGGGACCCCGACCAGGTCCGGGCGGGCGGCGTCCTCTCCAGCCAGTGTCTCTTCGGGAGCGCCGTGCGGGTCCTCGACCGCATCGTGGACGGCGGGCTGGCCGCACCGCCGATCGACCGGCTCGCGCTGACCGTGGACGGCGTGGGCACCGTCGTAAGTGGCTTCGATGCGCCCGCGCAGTCCATCGACCAGCGGGTGGCCTGCGCGGCCCTCATCGAGGACTTCGTGGCAGCGGGAGGAGACTTCCGCGTCGCGGACATCGACCCCGCCGGTCTCGACGTCCTCGCGGGGCACCACGACCTGACGATCGTGGCGACCGGCACGGGGGACCTCGGCCGGATGTTCGTCCGCGACGCGGTGCGCAGCCCGTACGACCGTCCGCAGCGCGCTCTAGCTGTCGCCTACGTGCACGGGCTGCTCCCGGACGAGACAGGTGCCGAACTCACCATCACGGTCGCCCCGGGTGTCGGCGAACTGATCACCCTGCCGGCGCTCACGACGACGGGCCCCTGCTCGATCCTGGTTTTCGAAGGCGTGCCCGGCGGACCGATGGACCGCTTCGAGGACGTGACCGGCCCCGAGGAGCACCTGCGCCGCTGCCAGAAGGTCCTTGAGGAGGTCTTCCCCGCCGTGGCCGCGCGCGCCCGTTCCGTCGAGCTGACCGACGAGGGCGCCGTCCTGCGCGGCCGCCTGACGCCGACCGTGCGGAAGCCGGTGGCGTACACCGCCTCGGGCCACCCCGTGCTCGGCCTGGCCGACGCGGTCGTGCTGAACGACCCGGTCACCGCGCAGGGCGCGAACAACGCGGTCCACGCCGCCGGCTCCTACCTGGACGCCGTCTTGCACCGGGGCGCCCGGCCCTTCGACGCCGAGTGGATGCACGAGACCTTCGAGTCGTTCTGGCGCTCCTGGGGCCGCTGGTCGGTCGCCTGGACCAACCGCGTCCTGGCCGGTCTCCCCGCGCACGTCGTCGACCTGGTCCCGGTCGCCGCCCAGGAGCCGGCGGTCGCCGCCCTCTTCGCCGCCGGGTTCGACGACCCCTCGACGCTGCACGACTGGTGGTTCGACCCGGGTGATGCCGAGCGCATGCTGGCGGCGGTGCGCGCCGCGGAGCGGAGCGTGGACCCGAGAGCCCTGCGCAACGCCTTCGGCCAGTACGCCACGGGCGTCACCGTGGTCACCACCCGAGCCCCCGACGGTCGCAAGATCGGCGTCACCGCCAACTCCTTCACCTCCGTGTCGCTGGACCCGCCCCTGATCGCGTGGTGCCCGGCCAAGAAGGCCCCTAGCCTGCCCGACCTGATCGCCGCCTCCCACTTCGCGGTCAACGTCCTGGCGGCGGACCAGCACGAGCTGAGCCGGCAGTTCGCCACCCCCGCTCCGGACAAGTTCGCGGATGTTGCGTACCGGGACGGCATCGCCGGAGTGCCGCTGCTGGAGCGGTCGATCGCCCGTTTCCAGTGCCGCACCGTCCAGCGGGTGGAGGCCGGGGACCACATCATCTTCATCGGCGAGGTCGAGCACTTCGACACCTCCGACGGGGCCCCCCTCGTGTTCCACTCCGGCGGCTACAGGATCGCCGCCGACCATCCGGACTTCGCC

**References**

1. Stover CK, Cruz V, Fuerst TR, Burlein JE, Benson LA, Bennett LT, et al. New use of BCG for recombinant vaccines. Nature Publishing Group. 1991(6326).

2. Yao K, Xu LQ, Wang FQ, Wei DZ. Characterization and engineering of 3-ketosteroid-∆1-dehydrogenase and 3-ketosteroid-9α-hydroxylase in *Mycobacterium neoaurum* ATCC 25795 to produce 9α-hydroxy-4-androstene-3,17-dione through the catabolism of sterols. Metab Eng. 2014;24:181-91.
